# Identification of a Riboflavin–Boric Acid Complex by Electrospray Ionization Mass Spectrometry

**DOI:** 10.1007/s12011-026-05110-9

**Published:** 2026-04-21

**Authors:** Danny H. Kim, Curtis D. Eckhert

**Affiliations:** 1 Department of Public Health, California State University, Fullerton, CA USA; 2 Department of Environmental Health Sciences, Fielding School of Public Health, University of California Los Angeles, Los Angeles, CA USA

**Keywords:** Boron, Riboflavin, Electrospray ionization mass spectrometry (ESI-MS), Boron complex, NAD, cADPR

## Abstract

Boric acid ingestion in children and adults has been shown to increase urinary riboflavin excretion (1), while high intakes of riboflavin reduce symptoms of boron toxicity in rats, guinea pigs, and chicks (2). Boric acid, the physiological form of boron, exhibits an affinity for molecules containing vicinal diols. Esterification of vicinal diols by boric acid has been reported for NAD^+^, the active form of vitamin B_3_, and cADPR, a signaling molecule derived from NAD^+^ that functions in the Ca^2+^ signaling pathway and oxidative stress (3, 4, 5). Riboflavin (vitamin B_2_) contains a ribityl side chain with vicinal diols, and although indirect evidence suggests an interaction with boron, direct evidence has been lacking. In this study, electrospray ionization mass spectrometry (ESI-MS) was used to investigate complex formation between riboflavin and boric acid. In negative-ion mode at pH 10.3, riboflavin produced a dominant ion at *m/z* 375.3, and the addition of boric acid resulted in a new signal at m/z 401.3, corresponding to a + 26 Da increase relative to riboflavin and assigned to the formation of a 1:1 riboflavin-boric acid complex through boron-diol esterification. This species accounted for approximately 8% of the riboflavin signal, indicating a weak yet measurable binding. These findings provide direct analytical evidence for riboflavin-boric acid complexation. Together with previously reported interactions in humans and animals, these findings highlight the need to define a safe range of boric acid/riboflavin intake, as both are beneficial, but excessive intake of one may impact the status of the other.

## Introduction

**Scheme 1:**
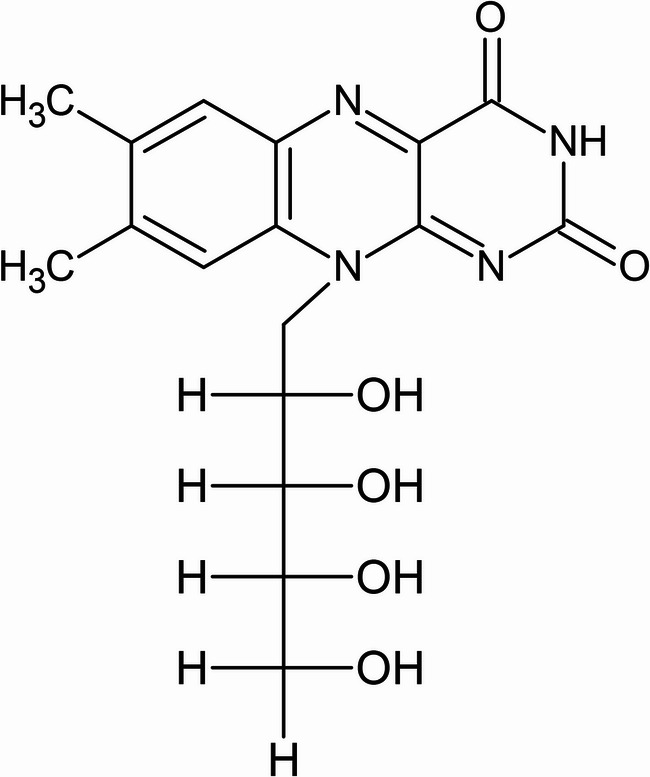
Structure of riboflavin illustrating the isoalloxazine ring and the ribityl side chain containing vicinal hydroxyl groups

Riboflavin is an essential water-soluble vitamin that serves as the biochemical precursor of flavin mononucleotide (FMN) and flavin adenine dinucleotide (FAD). FAD and FMN are coenzymes that bind tightly to flavoproteins and enable one- and two-electron transfer reactions. Flavoenzymes catalyze numerous redox reactions involved in energy production, erythrocyte synthesis, vitamin metabolism, and redox homeostasis in humans [6–8]. Free riboflavin is light sensitive and photolysis leads to the formation of lumachrome and lumiflavin. Excess dietary riboflavin has been reported in rats to damage the retina and cause a dose-dependent increase in autofluorescence and reduction of retinal outer segment height in rats [9]. Riboflavin deficiency disrupts mitochondrial function and fatty-acid metabolism, increases oxidative stress, and promotes apoptosis. It has been associated with anemia, diabetes, childhood neuropathy, and an elevated risk of cancer [6, 10, 11]. The redox process in riboflavin derivatives occurs exclusively on the isoalloxazine ring [12] whereas the ribityl side chain, which contains multiple hydroxyls (i.e., vicinal diols), enhances water solubility, contributes to enzyme binding, and provides the site for phosphorylation [13] (Scheme 1).

**Scheme 2:**
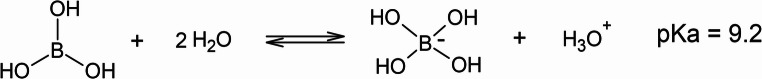
Dissociation equilibrium of boric acid in aqueous solution (pK_a_ = 9.2)

**Scheme 3:**
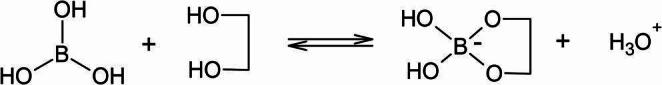
Formation of a cyclic boron esters (boroesters) between boric acid/borate and a diol molecule

Boron interacts with organic compounds that possess hydroxyl groups on adjacent carbon atoms, such as the *cis*-2,3-diol of ribose in nucleotides and the 1,2-diols of carbohydrates. As the fifth element in the periodic table, boron has three valence electrons and, under physiological conditions, occurs mainly as neutral boric acid, [B(OH)_3_], and its conjugate base, borate, [B(OH)_4_^−^], with an acid dissociation constant of approximately 9.2 at 25 °C (Scheme 2). Both species form reversible covalent complexes with compounds containing vicinal diols (Scheme 3).

In higher plants, boron functions as an essential micronutrient that contributes to cell-wall integrity, pollen-tube growth, and reproductive development [14]. Its biochemical role arises from its ability to crosslink polysaccharides that contain *cis*-diol residues, particularly rhamnogalacturonan II, through reversible diester formation [15]. The condensation of boric acid with a diol forms borate esters, and the equilibrium constant for this reaction depends strongly on pH (Scheme 3) [3, 16].

Boron is currently classified as beneficial to human health by the National Institute of Medicine [17]. Numerous studies have shown that it influences mineral metabolism, bone strength, cognitive performance, and immune function [18–21]. Nielsen [21] reported that a daily intake below 1 mg of boron may diminish its physiological benefits, suggesting that many of its biological effects involve the formation of boron esters (boroesters) with vicinal diols in biomolecules.

The purpose of this study is to detect and characterize the complexation between boric acid and riboflavin using electrospray ionization mass spectrometry (ESI-MS). While boron readily forms cyclic esters with *cis*-diols, such as those in ribose, is well established, its potential to associate with vicinal but non-*cis* diols has not been conclusively demonstrated in biologically relevant molecules by ESI-MS. Riboflavin provides a suitable model for this investigation because its ribityl moiety contains several adjacent hydroxyl groups capable of forming boroesters, yet lacks the rigid geometry of the ribose ring found in nucleotides. The results from this study allow comparison of the relative abundance of the riboflavin-boric acid complex with previously characterized nucleotide-boron complexes (NAD⁺/NADH) [3, 5], thereby clarifying how structural geometry affects boron binding as detected by ESI-MS. Accordingly, the present work seeks to confirm the formation of a riboflavin-boric acid complex under alkaline conditions using ESI-MS and (ii) to provide insight into how molecular geometry could affect boron complexation with hydroxyl-containing biological diols. Although this study does not seek to establish the occurrence of complexation under physiological conditions, it provides analytical evidence that riboflavin can associate with boron and presents a structural framework that helps explain why this interaction is substantially weaker than boron binding to ribose-containing nucleotides.

## Materials and methods

Enriched ^11^B(OH)_3_ (99.27% purity) was purchased from Eagle-Picher Technologies (Quapaw, OK, USA), and all other reagents and solvents were of analytical grade or higher. Ultrapure water containing < 10 nM boron was used for all the experiments. Ultrapure water was prepared by ion exchange treatment, and the boron content was checked by quantitative inductively coupled plasma mass spectrometry as described previously [22].

Sample preparation for ESI-MS analysis.

Water: acetonitrile: triethylamine mixtures (WAT), 50:50:2 (v: v:v), were used as a common solvent for flow injection analysis (FIA) of samples. To acquire an ESI mass spectrum of riboflavin, 200 µM riboflavin (pH 9.5) in WAT solvent was directly injected into the mass spectrometer. The riboflavin-boric acid complex was prepared by mixing boric acid (400 µM, final concentration) and riboflavin (200 µM, final concentration) solutions in WAT at room temperature. Samples dissolved in WAT were introduced (20 µl/injection) into a stream of the same solvent entering the ion source (10 µl/min).

### ESI-MS

Experiments were conducted on a PerkinElmer Sciex (Thornhill, Canada) API III triple quadrupole mass spectrometer equipped with an Ionspray source, which was tuned and calibrated in the positive ion mode as described previously [23]. The instrument resolution allowed for a 15–20% valley between the ^13^C-containing satellites of the polypropylene glycol/NH_4_^+^ singly charged calibrant ion at *m/z* 906. For analysis of riboflavin-boric acid complex, the instrument polarity was reversed and the ionspray voltage lowered to -3.5 kV. Solutions of samples were introduced by direct injection (20 µL/injection) into a stream of the same solvent entering the ion source (10 µL/min). Normal spectra were collected (profile mode) while the instrument was scanning a 100 *m/z* range (0.1Da step size, 6 ms dwell time, 6.66 s per scan, orifice − 60 V). Representative spectra were computed as the average of all the spectra accrued from each sample injection using instrument-supplied software (MacSpec, version 3.3, PE Sciex, Ontario, Canada).

## Results

**Fig. 1:**
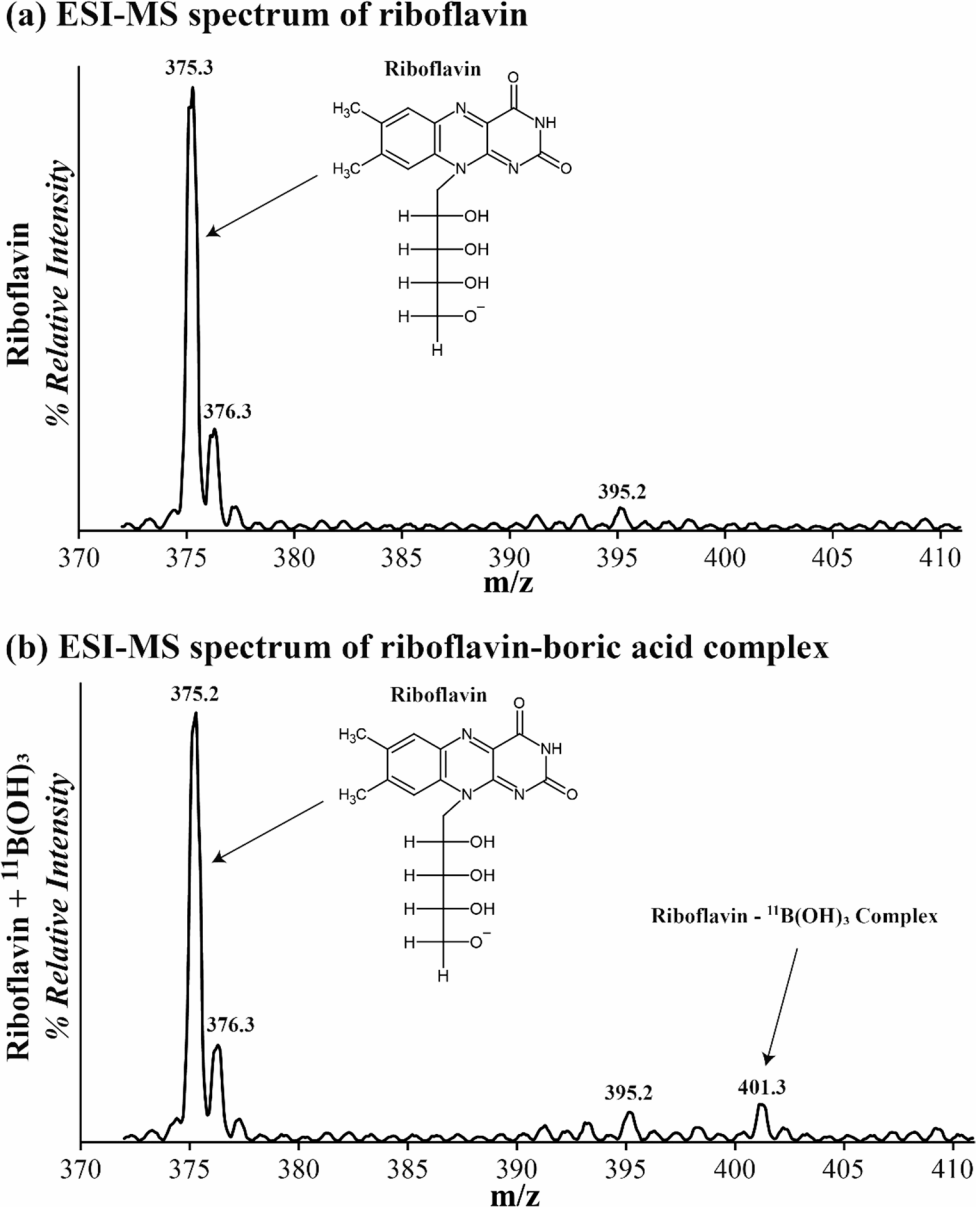
Negative ion ESI mass spectra of riboflavin and riboflavin-^11^boric acid mixture in WAT at pH 10.3. (**a**) 200 µM riboflavin showing an intense [M–H]^−^ signal at *m/z* 375.3 (calcd 375.1 Da). The signal at *m/z* 395.2 is assigned as an unknown impurity in the riboflavin sample. (**b**) A mixture of 200 µM riboflavin and 400 µM boric acid [^11^B(OH)_3_] produced a signal corresponding to a 1:1 riboflavin-^11^boric acid complex at *m/z* 401.3 (calcd 401.1 Da)

In WAT solvent at pH 10.3, negative-ion ESI-MS of 200 µM riboflavin produced a dominant molecular anion corresponding to the singly charged, alkali metal-free species [M–H]^−^ at *m/z* 375.3 (calcd 375.1 Da) (Fig. 1a). A smaller peak was also detected at *m/z* 395.2 (Δ 19.9 Da). Without further evidence, this signal was attributed to a minor impurity in the commercial riboflavin sample. When boric acid [^11^B(OH)_3_] was added to give a final concentration of 400 µM, the spectra revealed an additional signal at *m/z* 401.3. This ion was assigned to a singly charged 1:1 riboflavin–boric acid complex (calcd 401.1 Da for [M–H]^−^) (Fig. 1b).

The riboflavin signal at *m/z* 375.3 remained the predominant species in the spectrum, while the new signal at *m/z* 401.3 reached approximately 8% of the riboflavin signal intensity. The area ratio closely followed the relative intensity, indicating that only a small fraction of riboflavin formed the complex under these conditions. No higher-mass ions corresponding to riboflavin-borate species were detected. Although the riboflavin-boric acid signal is low in relative abundance, its reproducible appearance only upon addition of boric acid, together with the expected mass shift, is consistent with formation of a weak and reversible complex. The low intensity of this signal is therefore interpreted as a reflection of limited binding stability rather than analytical uncertainty.

**Fig. 2:**
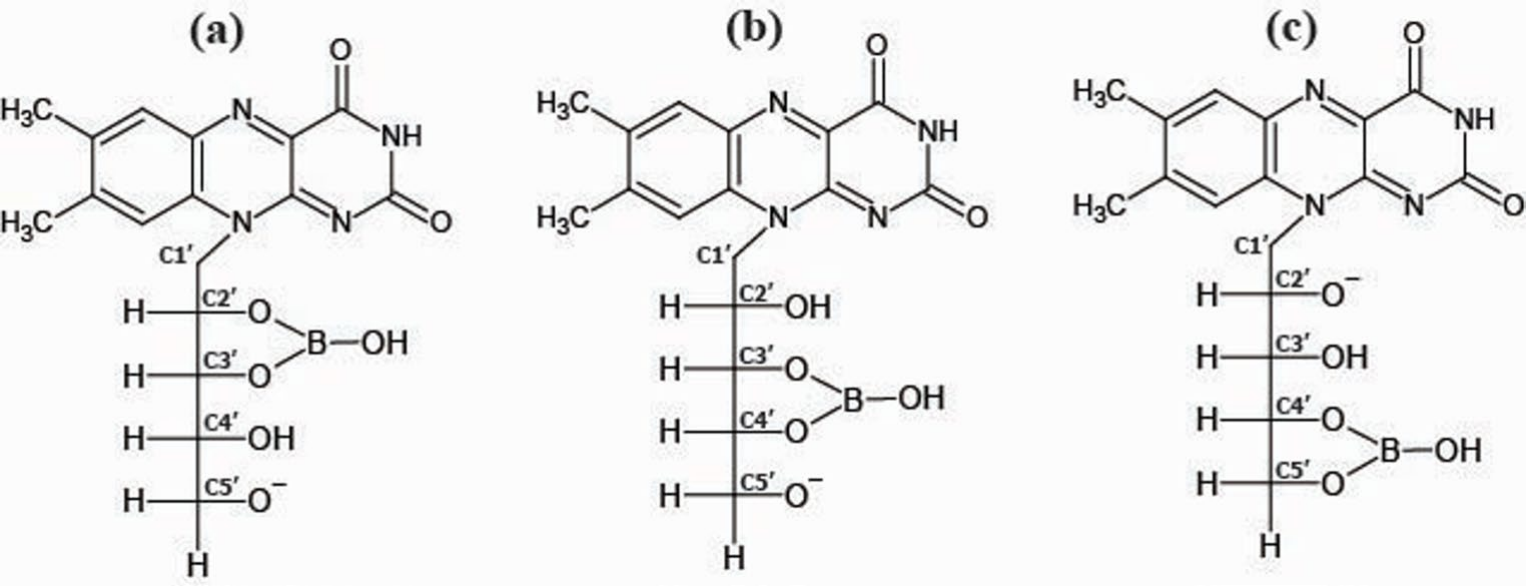
(**a**)–(**c**) Proposed configurations for riboflavin–boric acid complexes showing alternative binding sites on the ribityl side chain

The proposed structure of the riboflavin-boric acid complex is illustrated in Fig. 2. It likely involves coordination of boric acid with one of three possible vicinal diol sites on the ribityl side chain. Previous studies have shown that boron forms stable complexes with *cis*-diols in nucleotides [3] and sugar alcohols in plant extracts [24], and that the extent of complex formation depends on molecular charge and phosphorylation state [4]. In contrast to NAD^+^/NADH-boric acid complexation, the present study detected only the boric acid complex of riboflavin and no corresponding borate complex. If borate binding had occurred, a signal near *m/z* 427.3 would have been expected in the mass spectrum.

## Discussion

The interaction between riboflavin and boron has not been well studied, so a brief consideration of prior investigations is provided for context. A literature search using the keywords riboflavin and boron produced three papers of significance to the present study, all of which were published more than 50 years ago. The initial identification of an interaction between riboflavin and boric acid was discovered in the search for water-soluble riboflavin derivatives [25]. Frost observed that the addition of boric acid to aqueous mixtures of riboflavin with heating at 95^o^ increased the speed with which riboflavin was dissolved, but the solution was unstable upon cooling [25]. Heating was required to achieve solubility, and, for reasons not understood by the author, extended heat treatment at 95^o^ for 3 h produced solutions that remained stable for 12 months at both alkaline pH and pH 6.5. Solutions heated for 30 min were stable for 12 h, whereas those heated for 90 min remained stable for 7 days. Changes in positive optical rotation upon addition of boric acid to riboflavin provided evidence that the ribityl group participated in the solvent reaction. The inability of boric acid to solubilize the isoalloxazine group or riboflavin when the ribityl group was substituted supported this interpretation. The riboflavin-boron solution was shown to have full biological activity. Studies for acute and chronic toxicity in rats and dogs showed no deleterious effects on growth, estrus, or general well-being. The authors noted the absence of any signs of infection at the injection site when non-sterile syringes and needles were used in rat and dog experiments. This suggested the riboflavin-boron complex was “self-sterilizing”. This observation has been shown to be correct for boric acid. The major clinical use of boric acid is for the treatment of fungal and bacterial vaginitis (600 mg boric acid/day vaginal suppository). A retrospective study of 41 cases of microbial vaginitis reported an average cure rate of 76% for boric acid treatment of vulvovaginal candidiasis [26].

In blood, riboflavin exists in the free form bound to serum protein. It is converted into FMN and FAD in tissue. Roe and colleagues studied the interaction of dietary boric acid and riboflavin on growth in rats, guinea pigs, and chicks. High levels of dietary boric acid decreased growth, increased the excretion of riboflavin, and produced riboflavin deficiency symptoms [2]. Boric acid did not lower liver riboflavin concentrations where riboflavin exists as FMN and FAD. Increasing dietary riboflavin beyond requirements reduced the toxic effects of boron on growth and ameliorated deficiency symptoms. Evidence of an interaction between boric acid and riboflavin in humans was reported by the New York City Poison Control Center [1]. They measured urinary riboflavin excretion in 14 children and adults who had ingested boric acid. Boric acid ingestion was associated with large increases in riboflavin excretion within 24 h of ingestion in two-thirds of the patients. All three of these studies measured riboflavin and the riboflavin-boron complex using indirect methods, including bacterial and animal growth assays and ^12^C-riboflavin competitive binding assays.

The present study provides, to our knowledge, the first direct ESI-MS evidence for the formation of a detectable complex between riboflavin and boric acid. Upon addition of boric acid, a new ion at *m/z* 401.3 was observed, consistent with a 1:1 riboflavin-boric acid complex. The appearance of this additional signal indicates that boron can interact with the open-chain vicinal diols on the ribityl side chain of riboflavin, forming a neutral boric acid ester. Because the ribityl side chain contains three possible vicinal diol sites for boric acid complexation (Fig. 2), all of which would produce the same mass signal, ESI-MS alone cannot determine which site is involved. In this context, computational modeling may provide a useful complementary approach for identifying the energetically favored ribityl-diol binding site in future studies.

The formation of the riboflavin-boric acid complex is consistent with established boron chemistry. As a Lewis acid, boron is known to form covalent bonds with vicinal diols, producing cyclic borosters (Scheme 3) [24, 27, 28]. The observed mass increase of + 26 Da relative to the deprotonated riboflavin ion (*m/z* 375.3) corresponds to the addition of a single boric acid [B(OH)_3_] molecule, supporting complex formation with the ribityl diols.

Notably, no riboflavin-borate signal was detected. This finding parallels our previous observations in studies of boron complexation with NAD⁺ and NADH, in which boric acid, rather than borate, formed more prominent and stable ester complexes [3, 4]. The same preference is observed here, suggesting that boric acid is the more effective complexing species for vicinal diols. Even under alkaline conditions, ester formation may proceed preferentially through neutral boric acid, while the borate anion remains strongly hydrated and less favorable for direct coordination.

Boron speciation and boron-diol ester equilibria are strongly pH-dependent, and systematic evaluation across a physiological pH range would provide valuable insight into complex stability. Previous studies have demonstrated that boric acid forms stable, pH-dependent complexes with ribose-containing nucleotides, including NAD⁺ and NADH, in aqueous solution under physiological conditions, as established by both ESI-MS and ¹¹B nuclear magnetic resonance (NMR) spectroscopy [3, 4].These findings indicate that boron-diol complexation is not restricted to highly alkaline environments and further demonstrate that such complexes are not solely artifacts of the electrospray ionization process. Together, they provide a relevant framework for interpreting the present results.

**Fig. 3:**
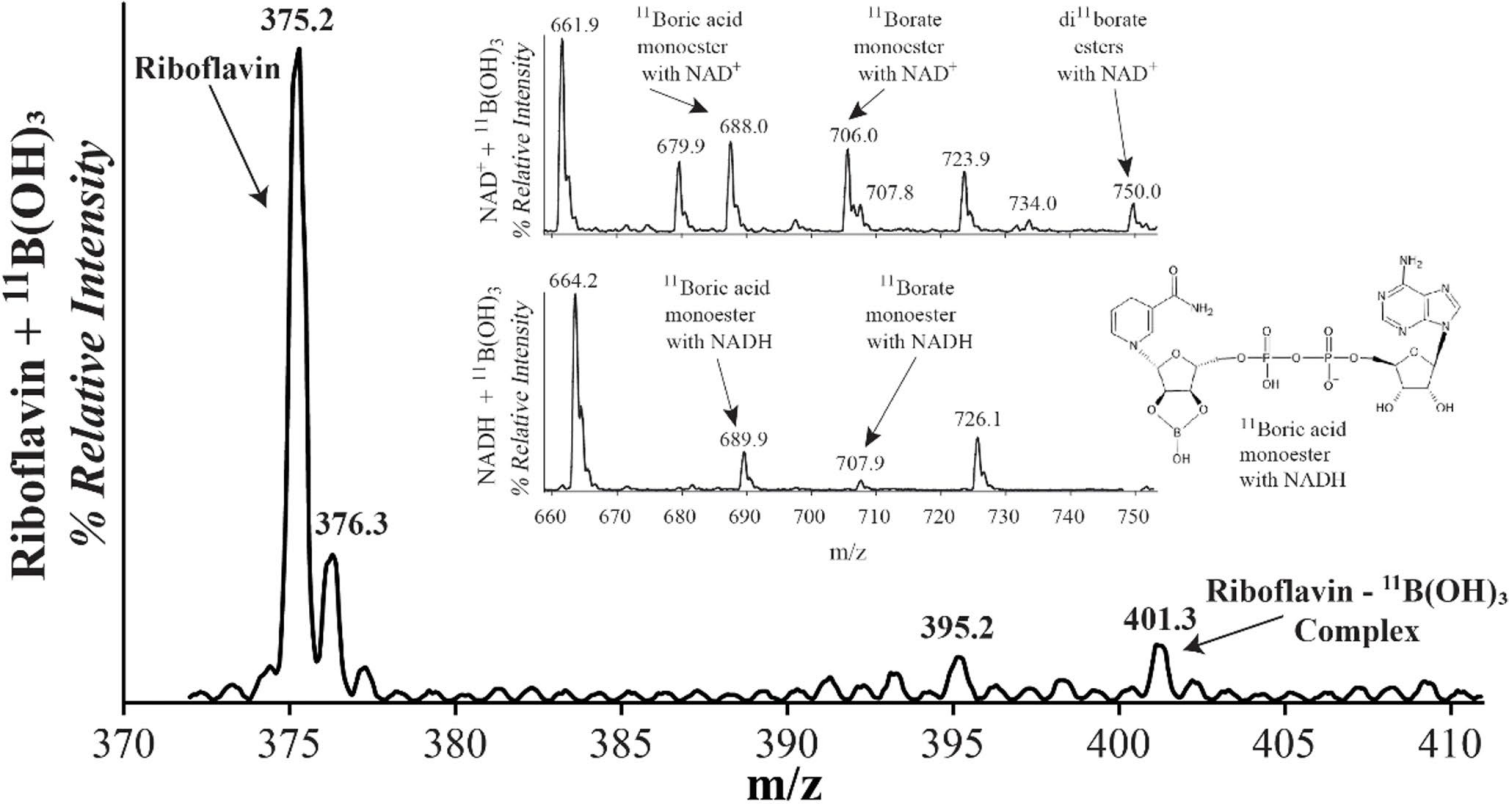
Negative-ion ESI-MS spectra showing formation of the riboflavin–boric acid complex and comparison with previously reported boron–nucleotide complexes. The main spectrum (bottom) shows riboflavin (*m/z* 375.2) and the riboflavin-^11^B(OH)_3_ complex (*m/z* 401.3) detected under alkaline conditions (200 µM riboflavin, 400 µM boric acid, pH 10.3). Insets (top) are reproduced from Kim et al. (2003) [3], showing boric-acid and borate monoesters and diesters with NAD⁺ and NADH (50 µM nucleotide, 50 µM boric acid, pH 10.3). Comparison illustrates that boron exhibits a stronger affinity for the *cis*-diol groups on ribose in NAD⁺/NADH than for the vicinal diols on the ribityl chain of riboflavin

Although the presence of three potential diol sites on the ribityl side chain might be expected to favor complexation, the relatively low intensity of the *m/z* 401.3 signal indicates that riboflavin forms a weak complex with boric acid under the experimental conditions. In earlier studies of NAD^+^ and NADH, boron formed stronger and more abundant complexes that were readily detectable by ESI-MS and ^11^B NMR spectroscopy, even at equimolar nucleotide-to-boron ratios [3]. This observation reflects the well-established preference of boron for *cis*-diols, such as those present in the ribose ring of nucleotides, where conformational constraint favors formation of a stable five-membered cyclic boroester [29]. In contrast, the riboflavin-boric acid interaction observed here is distinctly weaker, despite the higher boron ratio used in the experiment (1:2 riboflavin: boron) (Fig. 3). This difference underscores the importance of diol geometry, as rigid *cis*-diols promote stronger boron complexes, whereas the open-chain vicinal diols of the ribityl chain are more flexible and less favorable for forming a stable complex. Together, these observations highlight the role of diol configuration in determining the extent of boron-biomolecule association.

The weak riboflavin-boron complexation observed here also contrasts sharply with the highly stable boroester crosslinks found in plant cell walls, most notably in rhamnogalacturonan II. In plants, boron serves an essential structural role by forming diester bridges between specific *cis*-diol motifs, thereby stabilizing complex polysaccharide architectures required for cell wall integrity and growth [15]. In contrast, boron utilization in animals appears to rely on weaker, reversible interactions with small molecules containing vicinal diols, rather than on permanent structural crosslinks [30]. The preference for *cis*-diols could explain why boron associates more readily with nucleotides and plant polysaccharides than with open-chain polyols found in other biomolecules. Although the present study does not establish riboflavin-boron complexation under physiological conditions, the detection of weak and reversible complexation suggests potential biological implications that warrant future investigation. Transient boron association could, in principle, influence riboflavin stability or its accessibility for enzymatic conversion to FMN and FAD, without requiring formation of a stable, long-lived complex.

Several limitations of the present study should be noted. Nominal-mass ESI-MS allows detection of complex formation, but unambiguous molecular formula determination requires complementary high-resolution approaches. The weak and reversible nature of boron-diol interactions also limits the structural information that can be obtained from tandem MS. In addition, the experimental conditions were selected to facilitate analytical detection rather than to replicate physiological environments. Accordingly, ESI-MS is used here as a qualitative tool to establish the occurrence of boric acid complexation with riboflavin rather than to quantify binding strength. The markedly lower abundance of the riboflavin-boron complex relative to previously reported nucleotide-boron complexes is therefore interpreted in a comparative and mechanistic context, consistent with differences in diol geometry and conformational constraint, rather than as a direct measure of solution-phase affinity. These considerations do not detract from the primary conclusion that riboflavin forms a complex with boric acid, but they define the scope in which the results should be interpreted.

Within this context, future studies employing high-resolution mass spectrometry and isotopic pattern analysis could provide more definitive molecular formula assignment and improved characterization of low-abundance complexes. Additional work should also examine how factors such as pH, ionic strength, and boron speciation and prior heating affect the stability of the riboflavin-boron complex. Because the equilibrium between boric acid and borate anions depends strongly on pH, systematic ESI-MS and NMR experiments across a range of solution conditions could clarify whether complexation occurs under physiological environments. Comparative analyses involving other riboflavin derivatives, including FMN and FAD, as well as structurally related open-chain polyols, may help reveal how phosphate substitution and side-chain rigidity influence boron binding. It will also be important to examine whether boron complexation affects the kinetics of flavin-dependent enzymes, as such effects could provide valuable insights into the potential biochemical roles of boron-flavin interactions. Other areas to be explored are whether a riboflavin-boron complex occurs in blood, urine, and the large intestine.

## Data Availability

No datasets were generated or analysed during the current study.
